# Exploring the effect of plant nitrogen concentration on the nitrogen nutrition index of winter wheat under controlled irrigation conditions

**DOI:** 10.3389/fpls.2025.1609847

**Published:** 2025-07-01

**Authors:** Mingxia Wang, Ben Zhao, Xiaoli Niu, Wanqiang Chu, Guijun Lv

**Affiliations:** ^1^ School of Hydraulic Engineering, Yellow River Conservancy Technical University, Kaifeng, Henan, China; ^2^ College of Tobacco Science, Henan Agricultural University, Zhengzhou, Henan, China; ^3^ College of Agricultural Equipment Engineering, Henan University of Science and Technology, Luoyang, Henan, China

**Keywords:** water deficit, nitrogen status, tissue nitrogen concentration, component analysis, leaf area

## Abstract

**Introduction:**

The nitrogen nutrition index (NNI) of winter wheat decreased under water deficit conditions, primarily due to an increase in the critical nitrogen concentration (%N_c_) associated with a reduction in shoot biomass (SB). However, the effect of plant nitrogen concentration (PNC) on NNI under water deficit conditions remains unclear. This study aimed to: (1) determine whether significant differences in PNC and leaf nitrogen concentration (LNC) of winter wheat exist among different water treatments under controlled conditions; (2) analyze the reasons for changes in PNC and LNC under water deficit conditions; and (3) assess the stability of relationships between PNC and LNC, as well as between plant nitrogen accumulation (NAp) and leaf area index (LAI), across different water treatments.

**Methods:**

To address the above mentioned objectives, a series of rainout shelter experiments were conducted during the winter wheat growing seasons from 2018 to 2021.

**Results and discussion:**

The results indicated that water deficit treatments limited PNC and LNC values at specific growth stages of winter wheat under controlled conditions. However, such severe water deficits are unlikely to occur in typical field conditions; thus, PNC was not identified as the primary factor affecting NNI in field environments experiencing water deficit. Component analysis clarified the causes behind the decline in PNC and LNC. The decline in specific leaf area (SLA) and leaf biomass fraction (LBF) contributed to the decrease in PNC, with SLA accounting for more variation than LBF. Similarly, declines in both SLA and specific leaf nitrogen (SLN) led to reduced LNC, with SLN explaining more variation in LNC than SLA across different water treatments. LNC was jointly controlled by both PNC and the ratio of SLN to LBF. Furthermore, water deficit did not alter the proportional linear relationship between NAp and LAI, suggesting that the impact of water deficit on PNC and LNC is limited, which helps a better understanding of the factors contributing to the declination of NNI.

## 1 Introduction

Nitrogen (N) is one of the primary nutrient inputs in intensive agricultural production systems worldwide; however, it also significantly contributes to environmental pollution through surface runoff, gaseous emissions, and other pathways (Tyagi et al., 2022). Over the past five decades, numerous studies have reported a significant increase in nitrate concentrations in aquatic ecosystems, primarily driven by excessive N fertilization in intensive agricultural systems, particularly in China (Hou et al., 2023). Cui et al. (2010) reported that China accounted for approximately 38% of global N fertilizer consumption, with total N input in the wheat-maize cropping system in North China exceeding normal crop N requirements by nearly 600 kg N ha^-1^ annually. Additionally, the overuse of N fertilizers has led to decreased agricultural profitability for farmers (Lu et al., 2021). To address both economic and ecological challenges, it is essential to improve N fertilizer management practices based on crop demand. Such approaches may help reduce fertilizer inputs, mitigate N-related environmental risks, and enhance farmers’ income.

Optimized N fertilizer management fundamentally relies in a clear understanding of crop N status (Zhao et al., 2016). The nitrogen nutrition index (NNI) is widely regarded as an effective and quantitative method for diagnosing crop N status within the framework of optimized N management (Ata-Ul-Karim et al., 2017; Zhao et al., 2021). The calculation of NNI is based on the crop’s critical N concentration (%N_c_) dilution curve of the crop (Lemaire et al., 2019). To date, %N_c_ dilution curves have been established for several major crops, including wheat, maize, and rice (Ata-Ul-Karim et al., 2013; Plénet and Lemaire, 1999; Justes et al., 1994). The %N_c_ represents the minimum N concentration (%N) required to achieve maximum biomass accumulation and is typically expressed as a negative power function (Lemaire et al., 2021). The interspecific and intraspecific variability in the parameters of the N dilution curve across different regions has been widely reported in the literature (Lemaire et al., 2008). Recently, a Bayesian hierarchical model has been employed to assess uncertainty in fitted %N_c_ curves (Makowski et al., 2020). Moreover, Ciampitti et al. (2021) demonstrated that variations in the maize %N_c_ parameters A1 and A2, representing critical N concentration and SB, respectively, were statistically insignificant under different genotype × environment × management (G × E × M) scenarios, particularly when SB exceeded 5 t ha^-1^. Similarly, Yao et al. (2021) found no significant differences in the parameters of winter wheat %N_c_ curves across G × E × M scenarios. The theoretical foundation of the %N_c_ dilution curve, as developed by Lemaire et al. (2008), is based on two main principles: (1) the ratio of LB to SB decreases allometrically with increasing SB, due to higher %N in leaves and lower %N in stems; and (2) N is distributed non-uniformly within a dense canopy, with preferential allocation to upper well-lit leaves and active recycling from lower canopy layers. Due to its strong physiological relevance and theoretical robustness, the %N_c_ dilution curve has proven to be stable across different growth environments and genotypes within a given crop species (Ciampitti et al., 2021).

Recent studies have questioned the stability of the %N_c_ dilution curve, suggesting that it may be influenced by soil water deficit. This influence has been demonstrated by a downward shift in the %N_c_ curve under water deficit conditions in tall fescue and wheat (split-plot design and 160-300 data points, Errecart et al., 2014; randomized block design and 120 data points, Hoogmoed and Sadras, 2018). Understanding the effect of soil water status on the %N_c_ curve is critical, as the %N_c_ curve derived from well-watered conditions may overestimate crop N deficiency under water deficit conditions (Hoogmoed and Sadras, 2018). Errecart et al. (2014) proposed four potential mechanisms for the observed reduction in %N_c_ under water deficit conditions: (1) increased concentration of water-soluble carbohydrates, leading to a passive dilution of plant N concentration (PNC); (2) a differential response in the relationship between SB and PNC to water deficit; (3) lower growth rates under water deficit conditions require less N for metabolic processes; and (4) accelerated leaf senescence and increased N mobilization rates. However, contrasting evidences have been reported in crops such as maize, wheat, tall fescue, and sorghum, in which no significant differences in %N_c_ values were observed between rainfed and irrigated conditions (historical experiments from 1979 to 1985 with 150-200 data points by Kunrath et al., 2018; and from 1990 to 2016 with 460-470 data points by Ciampitti et al., 2021). Ciampitti et al. (2021) argued that the statistical methods used by Errecart et al. (2014) may have led to inaccurate estimations of %N_c_. They further contended that the accumulation of water-soluble carbohydrate under water deficit conditions is unlikely to coincide with increases in N-rich soluble compounds. Besides, soil water deficit has a limited effect on the allometric relationship between SB and PNC. Thus, whether the %N_c_ dilution curve is truly altered under soil water deficit conditions remains a subject of ongoing debate.

Currently, the most explicit conclusion is that NNI tends to be lower under water deficit conditions than under non-water deficit conditions at the same N treatment level (Kunrath et al., 2018; 2020). The NNI is calculated using PNC and %N_c_. Under water deficit conditions, the intermediate cause of reduced NNI is a decline in SB, which, according to the %N_c_ calculation model, results in an increase in %N_c_ (Zhao et al., 2025). An indirect contributor may be changes in PNC across different water treatments. PNC, defined as the ratio of plant N accumulation (NA_p_) to SB, reflects the N dilution process in plant. Previous studies have reported variations in NA_p_ and SB during crop growth under both rainfed and irrigated conditions, both of which are influenced by soil water deficit (Errecart et al., 2020). Furthermore, Zhao et al. (2025) demonstrated that the response of PNC to water availability is more complicated across different water levels under rainout shelter experiments. However, the reasons behind changes in PNC remain unclear across various water treatments, especially under severe soil water deficit conditions. Although some studies have documented N dilution in leaves during crop development (Yao et al., 2014a, b; Sieling and Kage, 2021), few have specifically investigated changes in leaf N concentration (LNC) across different water treatments. While considerable research has focused on the effects of water deficit on SB and the %N_c_ dilution curve, relatively limited attention has been given to the direct effects of water stress on PNC and LNC, particularly under controlled experimental conditions. The morphological and physiological factors governing changes in PNC and LNC remain unclear, as does the potential alteration of the relationship between PNC and LNC under water deficit conditions.

Therefore, we hypothesize that variations in PNC and LNC are driven by morphological or physiological factors across different water-N coupling treatments. The main objectives of this study were: (1) to determine whether PNC and LNC differ significantly across various water treatments under controlled conditions; (2) to identify the key factors contributing to changes in PNC and LNC under water deficit conditions; and (3) to evaluate the stability of the relationship between PNC and LNC, as well as between NA_p_ and leaf area index (LAI), across different water treatments. This analysis is essential for improving our understanding of how water deficit affects PNC and LNC, which is crucial for accurately estimating the NNI under water deficit conditions.

## 2 Materials and methods

### 2.1 Experimental design and indicators measurement

**Figure 1:**
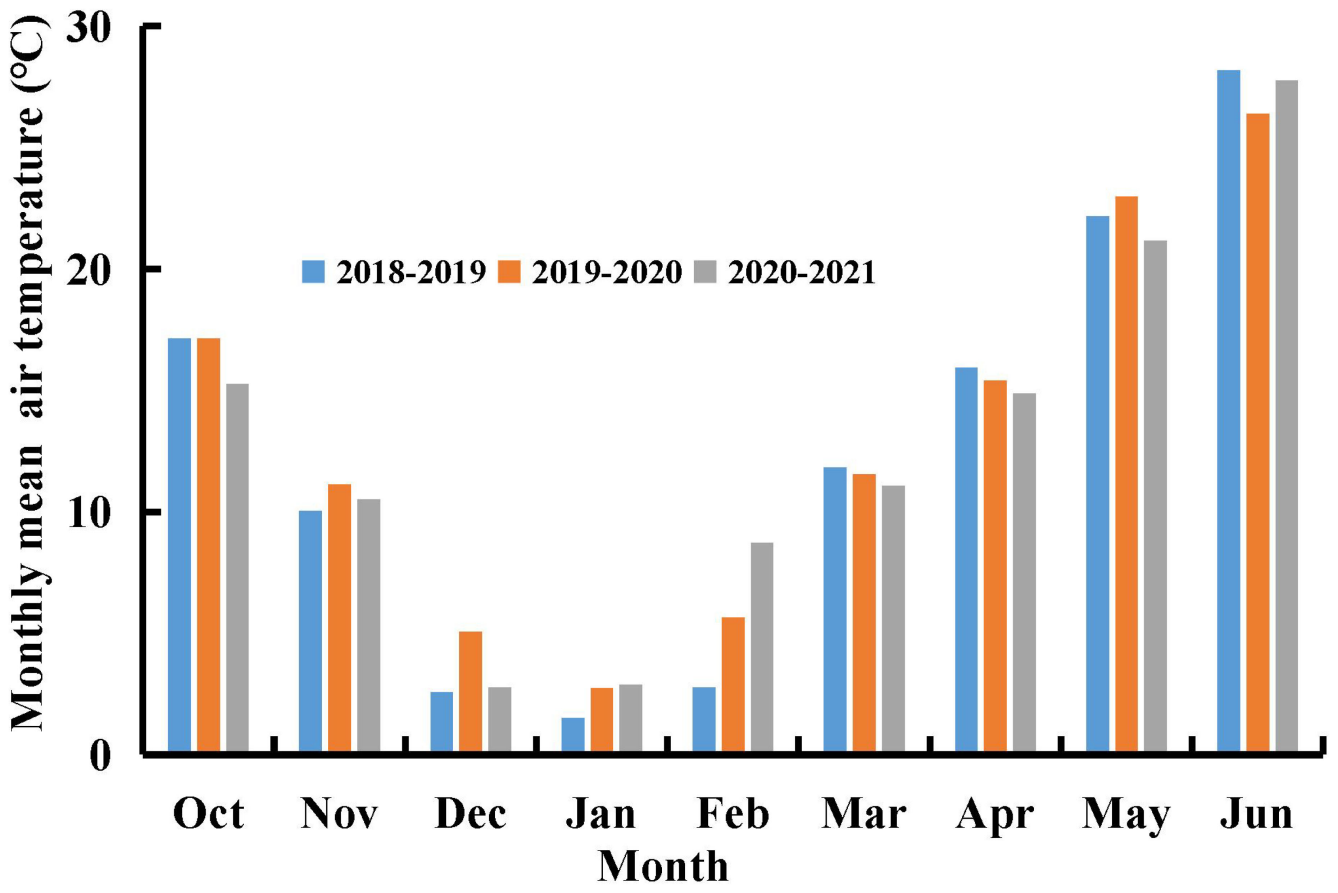
Monthly mean air temperature (°C) in 2018-2019, 2019-2020, and 2020-2021 growth seasons of winter wheat at Xinxiang, Henan Province, China

Three rainout shelter experiments on winter wheat were conducted in Xinxiang (35°18′N, and 113°52′E), Henan Province, China, during the 2018-2021 growing seasons. Each experiment included two N fertilizer treatments and four water deficit treatments. The rainout shelters effectively excluded natural rainfall from the experimental plots, allowing for controlled simulation of water deficit conditions. Irrigation was the sole water source for winter wheat throughout the experimental period. The monthly mean air temperatures in Xinxiang during the 2018-2021 winter wheat growing seasons are presented in **Figure 1**.

**Table 1** provides detailed information about the rainout shelter experiments conducted on winter wheat during the 2018-2021 growing seasons. Each plot within the rainout shelters covered an area of 6.67 m^2^. A total of 24 plots were established for each experiment, arranged in three replications. N fertilizer was assigned as the main plot factor with two levels: 225 kg N ha^-1^ (high N treatment, HN) and 75 kg N ha^-1^ (low N treatment, LN). These N rates represent typical low and high fertilization levels used in intensive winter wheat production systems in China (Cui et al., 2010). Half of the N fertilizer was applied as a basal dose before sowing, and the remaining half was applied as top-dressing at the stem elongation stage. Irrigation amount served as the split-plot factor, comprising four levels (W0-W3) designed to simulate varying degrees of soil water availability under controlled conditions. W0 represented a severe water deficit with no irrigation during critical growth stages, whereas W3 represented adequate water supply. These levels were determined based on previous studies in the North China Plain, where crop evapotranspiration ratios ranged from 40% to 100% (Gao et al., 2015; Zhao et al., 2025). This design enabled the evaluation of interactive effects between N application and water availability on plant N status under realistic agronomic conditions. Specific irrigation amounts and corresponding growth stages are listed in **Supplementary Table S1** (**Supplementary Material**). Drip irrigation was used throughout the growing period. Lines were positioned between wheat rows, with emitters spaced 20 cm apart and a flow rate of 2.2 L hour^-1^. Irrigation pressure was set to 0.1 MPa, and water usage for each event was measured using a calibrated water meter. Basal applications of phosphorus (150 kg P_2_O_5_ ha^-1^) and potassium (120 kg K_2_O ha^-1^) were applied uniformly across all treatments. No significant nutrient deficiencies, pest infestations, or disease occurrences were observed during the growing seasons. Abbreviations, units, and descriptions of all variables are provided in **Table 2**.

**Table 1:** Detailed information about the rainout shelter experiments.

Experiment		Site	Season	Cultivar	N treatment	Irrigation rate (mm)*	Sowing date	Soil type	Sampling stages	Actual evapotranspiration (mm)
Rainout shelter	1	Xinxiang	2018 -2019	Zhoumai 27	HN (225 kg N ha^-1^)	W0 (120)	16-Oct	Clay loam	Stem elongation	30.2
					LN (75 kg N ha^-1^)	W1 (210)			Booting	87.45
						W2 (300)			Anthesis	162.31
						W3 (390)			Grain filling	240.34
	2	Xinxiang	2019 -2020	Zhoumai 27	HN (225 kg N ha^-1^)	W0 (240)	15-Oct	Clam loam	Stem elongation	136.98
					LN (75 kg N ha^-1^)	W1 (330)			Booting	159.02
						W2 (420)			Anthesis	245.88
						W3 (510)			Grain filling	294.63
	3	Xinxiang	2020 -2021	Zhoumai 27	HN (225 kg N ha^-1^)	W0 (120)	18-Oct	Clam loam	Stem elongation	94.12
					LN (75 kg N ha^-1^)	W1 (210)			Booting	140.07
						W2 (300)			Anthesis	216.65
						W3 (390)			Grain filling	259.34

*Represents the total irrigation amount during the growth process of winter wheat.

**Table 2:** Description and units of the variables used in this paper.

Abbreviation	Variable	Unit
SB	Shoot biomass	t ha^-1^
LB	Leaf biomass	t ha^-1^
PNC	Plant nitrogen concentration	%
LNC	Leaf nitrogen concentration	%
%N_c_	Critical nitrogen concentration	%
N	Nitrogen	–
SLA	Specific leaf area	m^2^ kg^-1^
SLN	Specific leaf nitrogen	g m^-2^
LAI	Leaf area index	–
LBF	Leaf biomass fraction	%
NNI	Nitrogen nutrition index	–
NA_p_	Plant nitrogen accumulation	kg ha^-1^
NA_L_	Leaf nitrogen accumulation	kg ha^-1^

In the rainout shelter experiments (Exp. 1 to Exp. 3), a representative plant sample covering 0.36 m²was randomly collected from each plot at the stem elongation, booting, anthesis, and grain filling stages of winter wheat. The green leaf area of the sampled plants was measured using an LI-3000 meter (LI-COR), and the LAI was calculated by dividing the green leaf area by the corresponding land area. Each plant sample was separated into three parts: stem (including the leaf sheath), leaf, and spike. All samples were oven-dried at 70°C to a constant weight and weighed using an electronic balance to determine the dry biomass of each plant part. Subsequently, the dried samples were ground, passed through a 1 mm sieve, and stored in sealed bags for N concentration (%N) analysis using the Kjeldahl method. Leaf N accumulation was calculated by multiplying LNC by LB. Total SB and NA_p_ were determined by summing the biomass and N accumulations of the stem, leaf, and spike, respectively. The PNC was calculated as the ratio of NA_p_ to SB.

### 2.2 Plant nitrogen concentration and leaf nitrogen concentration, and their components

According to the findings of Lemaire et al. (2007 and 2008), the relationship between NA_p_ and LAI could be approximated as proportional during the vegetative growth period of the crop. This relationship was expressed as follows:


(1) $$NA_{\text{p}}\approx S\times LAI$$

where S is the amount of NA_p_ per unit of LAI.

Therefore, PNC could be expressed as follows:


(2) $$PNC=\frac{NA_{\text{p}}}{SB}\approx S\times \frac{LAI}{SB}$$

Equation 2 could be further decomposed into two primary components: (1) specific leaf area (SLA), defined as the ratio of LAI to LB, and (2) leaf biomass fraction (LBF), defined as the ratio of LB to SB. Therefore, PNC could be expressed by the following equation, i.e., Equation 3:


(3) $$PNC\approx S\times \frac{LAI}{SB}=S\times \frac{LAI}{LB}\times \frac{LB}{SB}$$

Leaf N concentration (LNC) was defined as the ratio of leaf N accumulation (NA_L_) to LB. Therefore, LNC could be expressed as follows Equation 4:


(4) $$LNC=\frac{NA_{L}}{LB}$$

Equation 4 could be further decomposed into two primary components: (1) specific leaf N (SLN), defined as the ratio of NA_L_ to LAI, and (2) specific leaf area (SLA), defined as the ratio of LAI to LB. Therefore, LNC could be expressed by the following equation Equation 5:


(5) $$LNC=\frac{NA_{L}}{LB}=\frac{NA_{L}}{LAI}\times \frac{LAI}{LB}$$

The relationship between PNC and LNC was derived by combining Equations 3 and 5, resulting in the following equation:


(6) $$LNC=\frac{1}{S}\times \frac{\frac{NA_{L}}{LAI}}{\frac{LB}{SB}}\times PNC=\frac{1}{S}\times \frac{SLN}{LBF}\times PNC$$

### 2.3 Component analysis

The component expressions of PNC and LNC were presented in Equations 3 and 5, respectively. Component analysis was employed to assess the net contribution of each variable, both directly and indirectly through its interaction with the other variable (Dordas, 2011). This analysis involved linearizing multiplicative relationships using a logarithmic transformation, followed by calculating the contribution of each component trait to the sum of squares of the resultant trait. The sum of the cross products between each component and the resultant trait ( $\sum x_{i}y_{i}$ ) was divided by the sum of squares of PNC and LNC ( $\sum y_{i}^{2}$ ) to determine the relative contribution of each component variable. The component expressions of PNC and LNC were analyzed as follows Equations 7 and 8:


(7) log(PNC)=log(SLA)+log(LBF)

(8) log(LNC)=log(SLA)+log(SLN)

### 2.4 Statistical analysis

After confirming the assumptions of normality and homogeneity of variance, a one-way ANOVA followed by Tukey’s HSD *post-hoc* test was conducted to evaluate differences in PNC and LNC among various water treatments under the same N levels and growth stage conditions. The significance level was set at *p* ≤ 0.05, 0.01, and 0.001, respectively. Allometric relationships (y=ax^b^) between PNC and LNC, LAI and NA_p_, and SLA and LBF were fitted across different water and N coupling treatments during the 2018-2021 seasons. All statistical analyses were performed using SPSS version 22 (SPSS Inc., Chicago, IL, USA).

## 3 Results

### 3.1 Changes in plant and leaf nitrogen concentrations from stem elongation to grain filling stages under different nitrogen and water coupling treatments

Plant nitrogen concentration (PNC) and LNC generally exhibited a decreasing trend from stem elongation to grain filling stage across N and water treatments in winter wheat (**Tables 3**, **4**). PNC was consistently higher under HN compared to LN at the same water treatment across all growth stages. The highest PNC value (2.76%) was recorded at stem elongation under HNW2 treatment in the 2019-2020 season, while the lowest (0.81%) was observed at grain filling stage under LNW0 in the 2020-2021 season (**Table 3**). LNC exhibited a similar trend, with the maximum value of 4.45% at booting stage under HNW2 treatment in the 2019-2020 season, and the minimum value of 0.43% at grain filling stage under LNW0 treatment in the 2020-2021 season (**Table 4**).

**Table 3:** Plant nitrogen concentration from stem elongation to grain filling across different nitrogen and water coupling treatments in the 2018 to 2021 seasons of winter wheat.

Experiment/ season	Growth stages	Treatment				P-value	*F* test
		HNW0	HNW1	HNW2	HNW3		
Exp. 1	Stem elongation	2.08	2.33	2.22	2.55	0.002	**
(2018-2019)	Booting	1.77	1.94	1.84	2.06	0.02	*
	Anthesis	1.55	1.66	1.6	1.73	0.22	ns
	Grain filling	1.5	1.52	1.45	1.64	0.16	ns
		LNW0	LNW1	LNW2	LNW3		
	Stem elongation	1.9	1.92	2.01	2.13	0.38	ns
	Booting	1.77	1.63	1.68	1.75	0.61	ns
	Anthesis	1.55	1.34	1.49	1.51	0.47	ns
	Grain filling	1.22	1.32	1.28	1.28	0.77	ns
		HNW0	HNW1	HNW2	HNW3		
Exp. 2	Stem elongation	2.59	2.74	2.76	2.72	0.77	ns
(2019-2020)	Booting	2.13	2.25	2.33	2.34	0.49	ns
	Anthesis	1.32	1.47	1.48	1.55	0.08	ns
	Grain filling	1.5	1.6	1.54	1.54	0.18	ns
		LNW0	LNW1	LNW2	LNW3		
	Stem elongation	1.54	1.57	1.47	1.57	0.41	ns
	Booting	1.41	1.42	1.4	1.4	0.96	ns
	Anthesis	1	1	0.93	0.97	0.58	ns
	Grain filling	1.05	1.05	1.07	0.94	0.67	ns
		HNW0	HNW1	HNW2	HNW3		
Exp. 3	Stem elongation	2.31	2.56	2.62	2.45	0.08	ns
(2020-2021)	Booting	1.39	1.64	1.7	1.57	0.0001	***
	Anthesis	1.39	1.53	1.58	1.45	0.02	*
	Grain filling	1.28	1.32	1.3	1.29	0.63	ns
		LNW0	LNW1	LNW2	LNW3		
	Stem elongation	1.29	1.72	1.95	1.64	0.008	**
	Booting	0.7	1.11	1.18	1.09	0.005	**
	Anthesis	0.87	1.31	1.23	1	0.013	*
	Grain filling	0.81	1.21	0.96	0.84	0.011	*

*significance at p<0.05; **significance at p<0.01; ***significance at p<0.001; ns, not significant.

**Table 4:** Leaf nitrogen concentration from stem elongation to grain filling across different nitrogen and water coupling treatments in the 2018 to 2021 seasons of winter wheat.

Experiment/ season	Growth stages	Treatments				P-value	*F* test
		HNW0	HNW1	HNW2	HNW3		
Exp. 1	Stem elongation	3.79	4.15	4.13	4.32	0.008	**
2018-2019	Booting	3.68	4.05	3.66	4	0.03	*
	Anthesis	3.48	3.79	3.56	3.64	0.08	ns
	Grain filling	2.78	3.37	3.51	3.51	0.0001	***
		LNW0	LNW1	LNW2	LNW3		
	Stem elongation	3.63	3.81	3.73	3.74	0.86	ns
	Booting	3.71	3.66	3.85	3.85	0.55	ns
	Anthesis	3.4	3.2	3.38	3.26	0.81	ns
	Grain filling	2.01	2.89	2.72	3.11	0.01	*
		HNW0	HNW1	HNW2	HNW3		
Exp. 2	Stem elongation	3.87	4.05	3.99	3.92	0.91	ns
2019-2020	Booting	4.1	4.31	4.45	4.39	0.35	ns
	Anthesis	3.04	3.59	3.54	3.55	0.02	*
	Grain filling	2.24	2.56	3.14	3.21	0.03	*
		LNW0	LNW1	LNW2	LNW3		
	Stem elongation	3.05	3.13	2.56	2.99	0.29	ns
	Booting	3.34	3.31	3.06	3	0.66	ns
	Anthesis	1.88	1.98	1.7	1.83	0.93	ns
	Grain filling	0.86	0.68	1.12	0.92	0.49	ns
		HNW0	HNW1	HNW2	HNW3		
Exp. 3	Stem elongation	4.41	4.44	4.32	4.28	0.79	ns
2020-2021	Booting	3.09	3.92	3.84	3.52	0.001	**
	Anthesis	3.12	3.68	3.72	3.59	0.02	*
	Grain filling	0.99	2	2.71	2.79	0.004	*
		LNW0	LNW1	LNW2	LNW3		
	Stem elongation	2.2	3.29	3.43	2.98	0.03	*
	Booting	1.74	2.57	2.85	2.67	0.02	*
	Anthesis	1.85	3.02	2.86	2.35	0.007	**
	Grain filling	0.43	0.91	1.01	0.57	0.02	*

* significance at p<0.05; ** significance at p<0.01; *** significance at p<0.001; ns,not significant.

Notably, PNC showed significant differences among water treatments under the same N level in 8 out of 24 combinations (33%), whereas LNC exhibited significant effects in 13 of the 24 combinations (54%). For example, PNC responded significantly to water treatments at stem elongation and booting stages during the 2018-2019 season under HN conditions (p< 0.05), and at multiple growth stages under LN treatments in the 2020-2021 season. In contrast, significant differences in LNC were more widespread, especially at later growth stages (e.g., during the grain filling stage under both HN and LN treatments across all three seasons). These findings suggested that LNC may be more sensitive than PNC to water availability, particularly during grain filling stage. The underlying mechanisms responsible for the differing responses of PNC and LNC to water treatments warrant further investigation.

Generally, both PNC and LNC declined with increasing water deficit, especially under low N conditions. However, the trends also indicated that high N input can partially mitigate the decline in tissue N concentrations under water deficit conditions.

### 3.2 Changes in leaf mass fraction, specific leaf area, and specific leaf nitrogen from stem elongation to grain filling stage under different nitrogen and water coupling treatments

**Figure 2:**
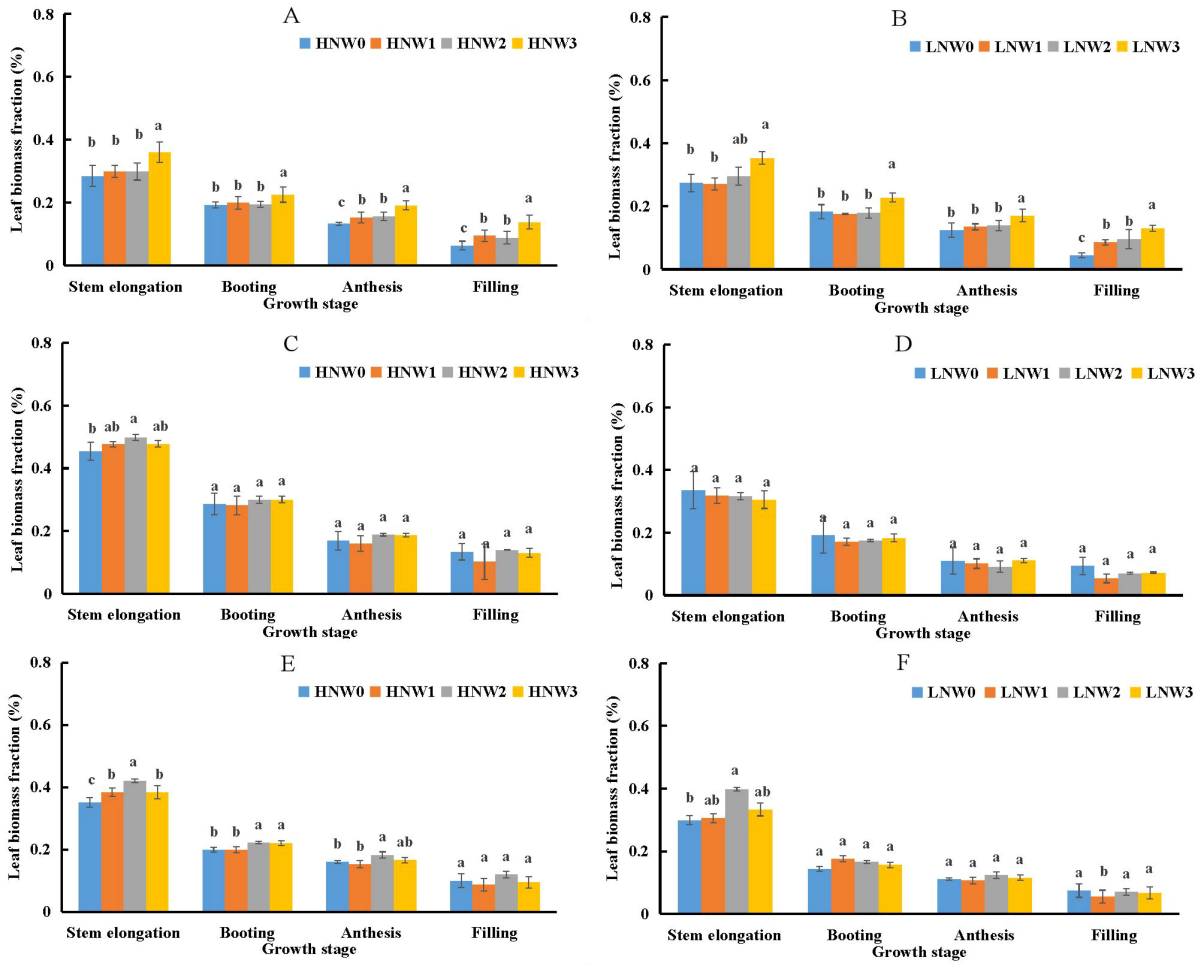
Changes in leaf biomass fraction from stem elongation to grain filling in winter wheat across different nitrogen and water coupling treatments **(A)** 2018-2019 HN; **(B)** 2018-2019 LN; **(C)** 2019-2020 HN; **(D)** 2019-2020 LN; **(E)** 2020-2021 HN; **(F)** 2020-2021 LN. Different letters indicate significant differences among treatments at the 5% probability level according to Tukey’s HSD post-hoc test.

As given in Equation 3, LBF was identified as the primary contributing component to PNC. LBF significantly declined from stem elongation to grain filling stage in winter wheat (**Figure 2**). The highest LBF value (0.5) was observed under HNW2 treatment at the stem elongation stage, while the lowest value (0.05) was recorded under LNW1 treatment at grain filling stage during the 2019-2020 season, reflecting a 10-fold difference between the two extremes. In the 2018-2019 season, LBF values under HN and LN treatments were generally similar at the same water conditions and growth stages. However, during the 2019-2020 and 2020-2021 seasons, LBF values under HN treatments were consistently exceeded by those under LN treatments at the same water level. Irrigation amounts also influenced LBF, which tended to decrease from mild to severe water deficit treatments under the same N treatments. This trend indicated that a smaller proportion of SB was allocated to leaf organs under severe water deficit conditions.

**Figure 3:**
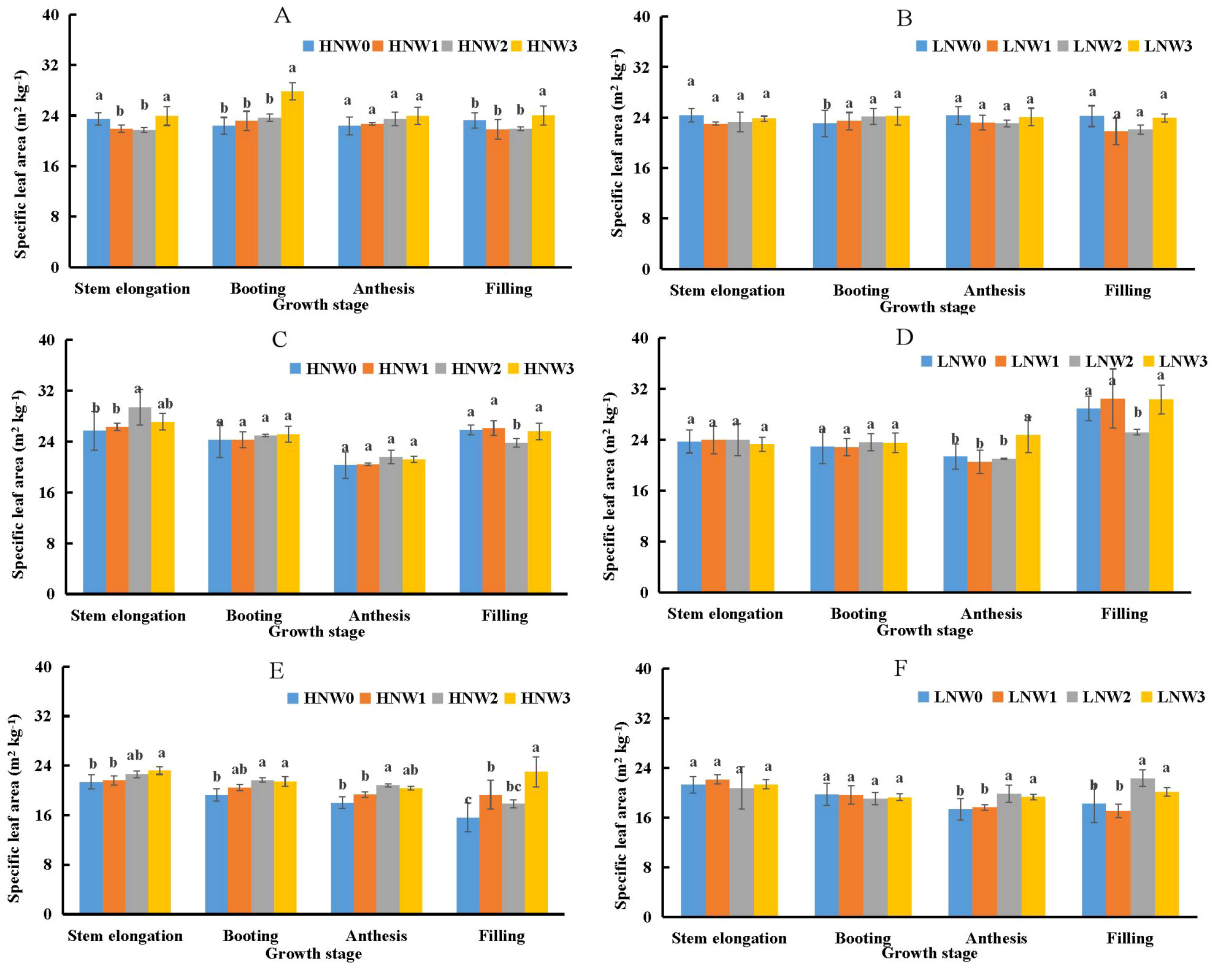
Changes in specific leaf area from stem elongation to grain filling in winter wheat across different nitrogen and water coupling treatments **(A)** 2018-2019 HN; **(B)** 2018-2019 LN; **(C)** 2019-2020 HN; **(D)** 2019-2020 LN; **(E)** 2020-2021 HN; **(F)** 2020-2021 LN. Different letters indicate significant differences among treatments at the 5% probability level according to Tukey’s HSD post-hoc test.

Specific leaf area (SLA) was identified as the second most important component influencing PNC (Equation 3) and the primary component influencing LNC (Equation 5). SLA showed a slight decreasing trend from stem elongation to anthesis, followed by an increase from anthesis to grain filling stage across most N and water treatment combinations in winter wheat during the 2018-2021 growing seasons (**Figure 3**). The maximum SLA value of 30.31 m^2^ kg^-1^ was observed under LNW3 treatment at grain filling stage in the 2019-2020 season, while the minimum value of 15.61 m^2^ kg^-1^ was recorded under HNW0 treatment at the same stage in the 2020-2021 season, representing a 2-fold difference between these extremes. Generally, there were no significant differences in SLA between HN and LN treatments under the same water conditions at most growth stages. Although SLA was significantly lower under severe water deficit conditions compared to mild or non-severe deficit conditions under the same N treatment in certain growth stages, the absolute differences in SLA values across water treatments remained relatively small before grain filling stage. The largest difference was observed at grain filling stage under HN conditions during the 2020-2021 season (**Figure 3E**), where SLA differed by 7.4 m^2^ kg^-1^ between the W3 and W0 treatments.

**Figure 4:**
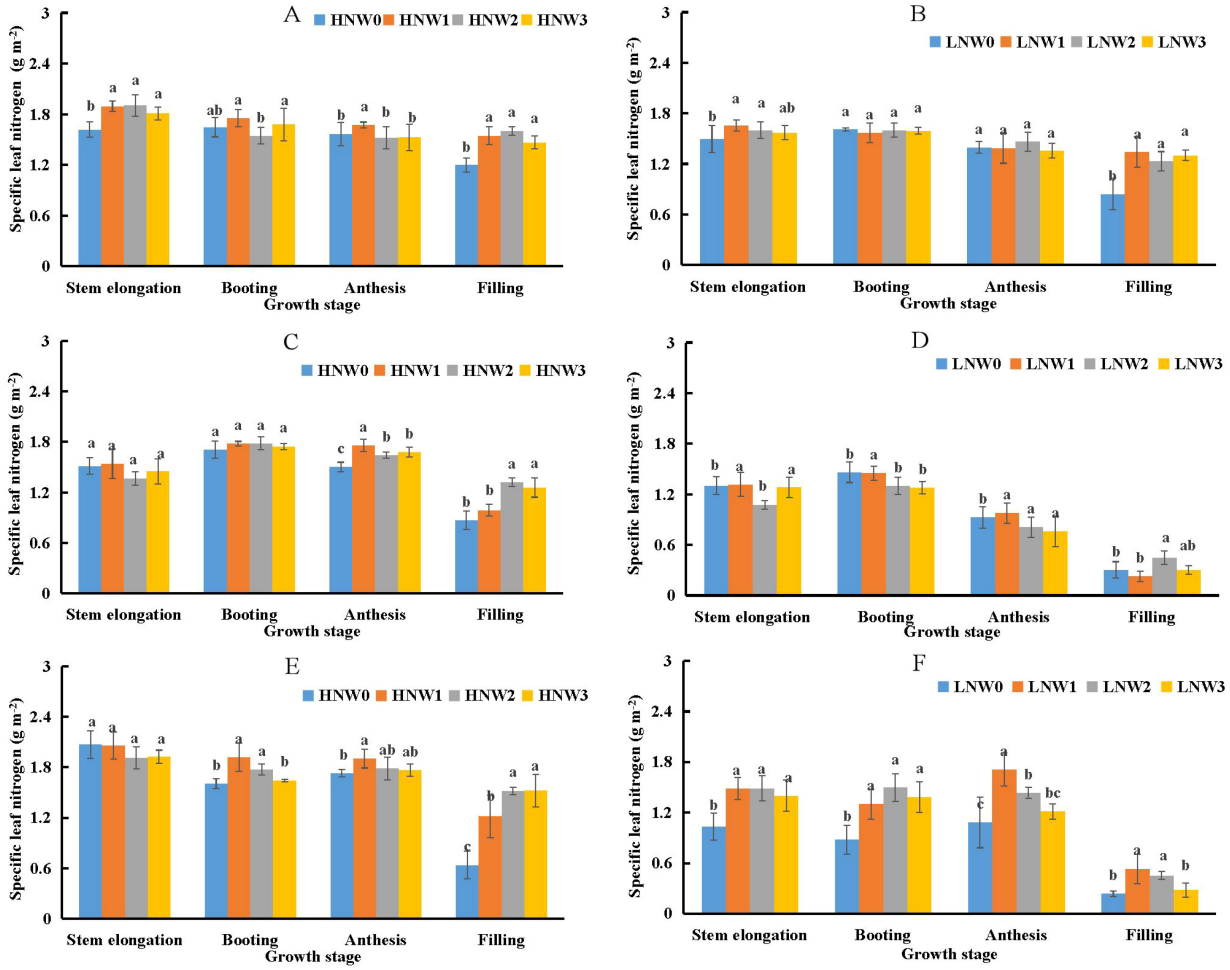
Changes in specific leaf nitrogen (SLN) from stem elongation to grain filling stage in winter wheat across different nitrogen and water treatment combinations **(A)** 2018-2019 HN; **(B)** 2018-2019 LN; **(C)** 2019-2020 HN; **(D)** 2019-2020 LN; **(E)** 2020-2021 HN; **(F)** 2020-2021 LN. Different letters indicate significant differences among treatments at the 5% probability level according to Tukey’s HSD post-hoc test.

Specific leaf nitrogen (SLN) was identified as another important component influencing LNC using the component analysis given in Equation 5. SLN remained relatively stable from stem elongation to anthesis but declined sharply from anthesis to grain filling stage under the same N and water coupling treatments (**Figure 4**). The maximum SLN value (1.93 g m^-2^) was recorded under HNW3 treatment at stem elongation stage during the 2020-2021 season. In contrast, the minimum SLN value (0.2 g m^-2^) was observed under LNW0 treatment at grain filling stage in the 2019-2020 season, representing nearly a 10-fold difference. SLN values under HN treatment were generally higher than those under LN treatment at the same water level across all treatments from the 2018-2021 growing seasons. In some growth stages, significant differences in SLN were observed between severe and non-severe water deficit treatments under the same N condition. However, at other stages, SLN values were relatively similar between the two water deficit levels under the same N treatment. For example, SLN values remained comparable from stem elongation to anthesis under LN treatment during the 2018-2019 growing season (**Figure 4B**), and at stem elongation and booting stages under HN treatment during the 2019-2020 season (**Figure 4E**). The largest observed difference occurred at grain filling stage under HN treatment in the 2020-2021 growing season (**Figure 4E**), where SLN differed by 0.88 g m^-2^ between the W3 and W0 treatments.

### 3.3 Component analysis of plant and leaf nitrogen concentrations under different nitrogen and water coupling treatments

The relative contributions of the component factors to PNC and LNC are presented in **Table 5**. According to Equation 7, SLA contributed more to the variation in PNC than LBF across all N and water coupling treatments during the 2018-2021 growing seasons. Similarly, based on Equation 8, SLN contributed more to the variation in LNC than SLA across all treatments during the same period. The maximum contribution of SLA to PNC (2.28) was observed under LNW0 treatment in the 2020-2021 growing season, while the minimum (1.56) was also recorded under LNW0 treatment, but in the 2018-2019 growing season. For LNC, the highest contribution of SLN (2.08) occurred under HNW3 treatment in the 2018-2019 growing season, whereas the lowest (1.66) was noted under LNW0 treatment in the 2020-2021 growing season. These variations in component contributions highlight significant interactions among N levels, water deficit treatments, and growing seasons in determining PNC and LNC.

**Table 5:** Contribution of each component trait to plant nitrogen concentration and leaf nitrogen concentration across different nitrogen and water coupling treatments in the 2018 to 2021 seasons.

Treatment	Y1 (Plant N concentration)						Y2 (Leaf N concentration)					
	X1 (Leaf biomass faction)			X2 (Specific leaf area)			X2 (Specific leaf area)			X3 (Specific leaf N)		
	2018- 2019	2019- 2020	2020- 2021	2018- 2019	2019- 2020	2020- 2021	2018- 2019	2019- 2020	2020- 2021	2018- 2019	2019- 2020	2020- 2021
HNW0	-0.71	-0.71	-0.99	1.71	1.71	1.99	-0.94	-0.92	-0.77	1.94	1.92	1.7
HNW1	-0.81	-0.65	-0.95	1.81	1.65	1.95	-0.95	-0.95	-0.88	1.95	1.95	1.88
HNW2	-0.94	-0.62	-0.83	1.94	1.62	1.83	-1.03	-0.97	-0.91	2.03	1.97	1.91
HNW3	-0.66	-0.65	-0.91	1.66	1.65	1.91	-1.08	-0.97	-0.93	2.08	1.97	1.93
LNW0	-0.56	-1.12	-1.28	1.56	2.12	2.28	-0.91	-0.79	-0.66	1.91	1.79	1.66
LNW1	-0.89	-1.15	-1.02	1.88	2.15	2.02	-0.92	-0.79	-0.75	1.92	1.79	1.75
LNW2	-0.87	-1.14	-1.06	1.87	2.14	2.06	-0.93	-0.79	-0.78	1.93	1.79	1.78
LNW3	-0.73	-1.19	-1.17	1.73	2.19	2.17	-0.94	-0.81	-0.72	1.94	1.81	1.72

### 3.4 Relationships between plant and leaf nitrogen accumulations and leaf area index across different nitrogen and water coupling treatments

**Figure 5:**
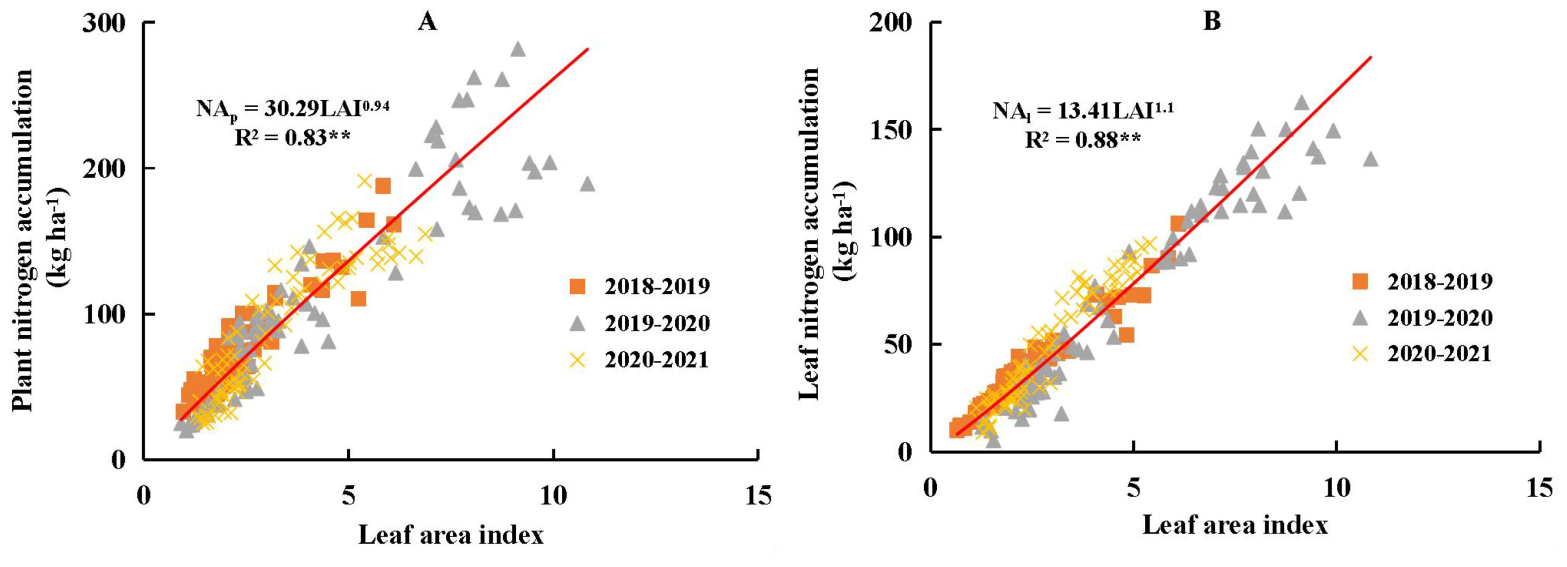
Relationship between plant nitrogen accumulation (NA_P_, **A**), leaf nitrogen accumulation (NA_L_, **B**) and leaf area index (LAI) during the vegetative period of winter wheat across different nitrogen and water coupling treatments in 2018-2021 growing seasons. ** significance at p<0.01.

Plant N accumulation (NA_P_) and NA_L_ both exhibited significant positive linear relationships with LAI across different N and water coupling treatments (**Figure 5**). Data collected under severe water deficit conditions were also included in **Figure 5**. Soil water availability did not affect the stability of the linear relationships among NA_P_, NA_L_, and LAI. The parameter *a* value was higher for the NA_P_-LAI relationship (30.29) than for the NA_L_-LAI relationship (13.41), whereas the *b* value was slightly lower for NA_P_-LAI (0.94) than for NA_L_-LAI (1.10). Overall, allometric patterns among NA_P_, NA_L_, and LAI were not strongly evident under different N and water coupling treatments during the vegetative growth period of winter wheat, thereby validating the assumption in Equation 1.

### 3.5 Relationships between leaf nitrogen concentration and plant nitrogen concentration, and between specific leaf nitrogen and leaf biomass fraction under different nitrogen and water coupling treatments

**Figure 6:**
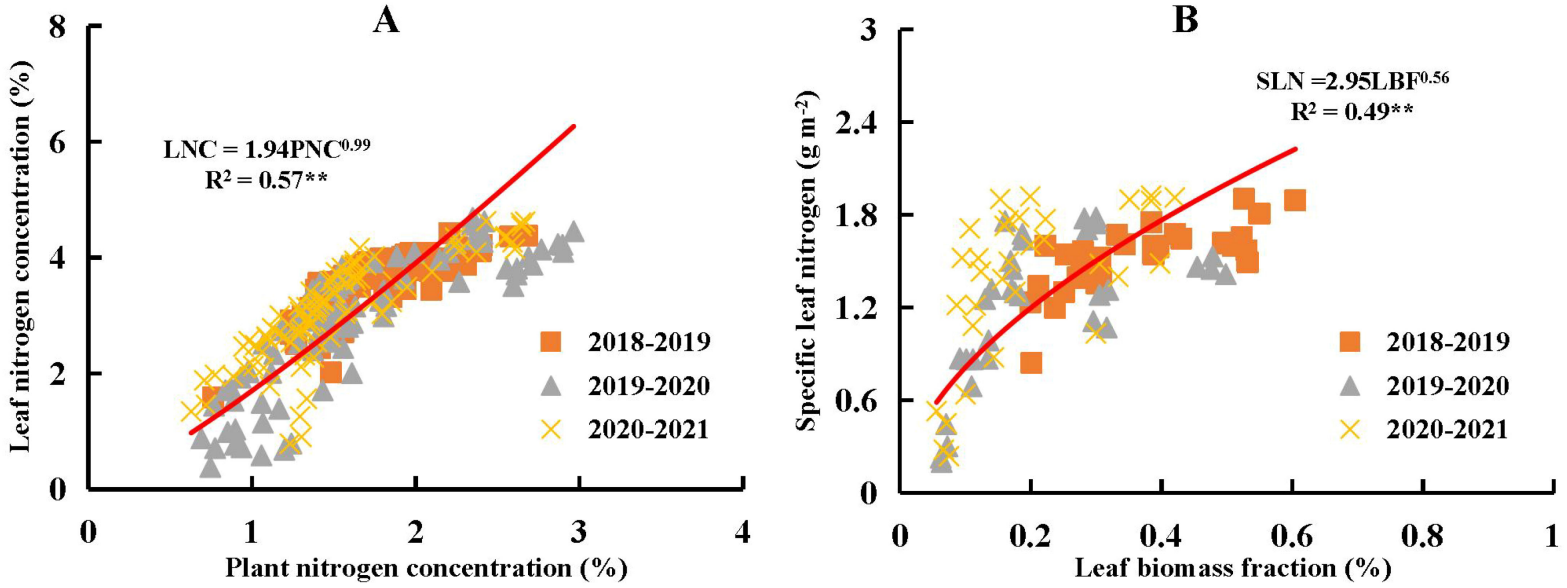
Relationship between plant nitrogen concentration (PNC) and leaf nitrogen concentration (LNC) as given in **(A)**, and relationship between specific leaf nitrogen (SLN) and leaf biomass fraction (LBF) as given in **(B)** across different nitrogen and water coupling treatments in the 2018-2021 growing seasons of winter wheat. ** significance at *p*<0.01.

Leaf N concentration (LNC) exhibited a significant positive linear relationship with PNC (**Figure 6A**), with a slope *b* of 0.99. This indicated that the decline in LNC was directly proportional to the decline in PNC from stem elongation to grain filling stages across different N and water coupling treatments during the 2018-2021 growing seasons. According to the results derived from Eq. (6), the intercept parameter *a* was 1.94, which is associated with both LBF and SLN. The relationship between LBF and SLN also demonstrated a significant positive allometric trend during the same developmental period and treatment conditions. The value of the intercept *a* reflected the trade-off between LBF and SLN.

## 4 Discussion

### 4.1 Effect of plant nitrogen concentration on the calculation of nitrogen nutrition index

The significant differences between PNC and LNC were observed under water deficit and N coupling treatments in controlled conditions (**Tables 3**, **4**). These differences were mainly found between severe and non-severe water deficit treatments. The severe water deficit treatment (W0) was characterized by the absence of irrigation from stem elongation to grain filling, with rainfall excluding using a rainout shelter (**Supplementary Table S1**). In cases where PNC showed significant differences, the ratio of actual evapotranspiration under the W0 treatment to the maximum evapotranspiration under the W3 treatment was less than 40% during the 2018-2021 growing seasons (Zhao et al., 2025). The use of the rainout shelter induced a more extreme water deficit during winter wheat growth, which likely contributed to the larger differences in PNC and LNC among the water treatments.

The severe water deficit was artificially imposed under rainout shelter conditions; however, such conditions were unlikely to occur under typical field conditions in normal agricultural production systems. Ji et al. (2015) reported that the 30-year average effective precipitation during the entire growth period of winter wheat in the same research region ranged from 75.2 mm to 177.5 mm, with an average value of 114.9 mm, accounting for 47.1% of the annual mean effective precipitation. Gao et al. (2015) reported a rainfall variation of 104 mm during the winter wheat growing season in the same region under rainfed conditions (0 mm irrigation) and noted that yield significantly declined from 7690 kg ha^-1^ to 2890 kg ha^-1^ when the ratio of actual to maximum evapotranspiration was 61%. These findings suggested that an evapotranspiration ratio as high as 61% could severely limit winter wheat yield. According to Ciampitti et al. (2021), the relationship between NA_p_ and SB, as well as the parameters of the %N_c_ curve, remained stable when the evapotranspiration ratio exceeded 40%. Consistently, our results also indicated that moderate and mild water deficits had minimal effects on PNC and LNC compared with sufficient water supply.

Our insights gained from this controlled scenario provided a valuable reference for understanding the thresholds at which N dilution patterns were significantly altered. In practical settings, mild to moderate water deficits were more common, and our results indicated that under such conditions, PNC and LNC remained relatively stable, suggesting that traditional interpretations of the NNI remain valid. However, in regions prone to episodic drought, even transient water stress may influence N dynamics. Therefore, integrating dynamic water status indicators into NNI models could improve the accuracy of N diagnostics under variable field conditions. These findings could facilitate improving irrigation and fertilization practices within the precision agriculture frameworks.

### 4.2 Analysis of the decline in plant nitrogen and leaf nitrogen concentrations

Component analysis was employed to analyze the underlying causes of the decline in PNC and LNC across the different water-N coupling treatments. Initially, PNC was separated into two main components. The first component, LBF, represented the ratio between LB and SB. The decline of LBF during the growth of winter wheat has been identified as a major driver of plant N dilution in plants (Lemaire et al. 2007 and 2008). Furthermore, LBF exhibited a declining trend with the intensification of the water deficit. Ratjen et al. (2016) reported similarly that LBF of winter wheat decreased under water deficit conditions, as plants tended to allocate less biomass to leaves and more to roots to enhance water uptake from deeper and broader soil layers. However, component analysis revealed that variation in SLA (the second component) explained a greater proportion of the variation in PNC among water treatments than did LBF (**Table 5**). This could be attributed to the fact that SLA, a morphological trait reflecting the ratio of leaf area to leaf dry mass, was more sensitive to water status and structural adjustments under water deficit conditions. Under such conditions, leaves generally became smaller and thicker due to reduced cell expansion and increased tissue density, resulting in a decline in SLA. These morphological changes could rapidly reduce leaf area, whereas leaf dry biomass tended to decrease more slowly, thereby intensifying the effect on SLA. Poorter et al. (2012) used the plasticity index to demonstrate that SLA was more sensitive to water availability than LBF. Similarly, Brisson and Casals (2005) showed that SLA was higher under irrigation compared to rainfed conditions after stem elongation in winter wheat. In contrast, LBF, which characterized biomass partitioning between leaves and shoots, changed more gradually and was largely influenced by long-term developmental allocation patterns.

The stronger contribution of SLA to PNC was consistent with previous studies that have highlighted its greater plasticity compared to LBF under water deficit conditions (Poorter et al., 2012). Poorter et al. (2010) presented a general response curve for scaled SLA, demonstrating a significant positive relationship between SLA and soil water availability, as evidenced by a positive slope value. Although water deficit tended to affect both LBF and SLA in a similar direction, moderate and mild water deficits had minimal impact on either trait (**Figures 2** and **3**; Poorter et al., 2010), which was insufficient to induce a significant decline in PNC. These findings suggested that morphological leaf traits, particularly SLA, may play a more immediate role in regulating N concentration dynamics under water deficit conditions. By quantifying the contributions of these components, our study confirmed that SLA was a key driver of N dilution patterns, especially during later growth stages such as grain filling, when structural changes in leaf became more pronounced.

In this study, LNC was separated into two main components. The first component was SLA, which also represented the first component of PNC. SLA collectively influenced the changes in both PNC and LNC; however, component analysis revealed that the decline in SLA explained less variation in LNC across different water treatments compared to PNC (**Table 5**). The observed decrease in SLA after anthesis was primarily due to a more rapid decline in leaf area relative to LB. As the plant transitioned into the grain filling stage, leaf senescence and reduced expansion led to a significant loss of green leaf area, whereas the reduction in dry leaf biomass occurred more gradually. This disproportionate change resulted in a decline of SLA. The second component was SLN, which accounted for a greater proportion of the variance in LNC. Lemaire et al. (2008) reported that the critical SLN value remained relatively stable across different species, ranging from 1.8 to 2.0 g m^-2^. In contrast, our results showed a broader range of SLN values across different water and N coupling treatments (**Figure 4**). SLN consistently declined with increasing water deficit. This decline during grain filling stage was primarily driven by N remobilization from leaves to grains, a key physiological process in wheat supporting grain development and protein synthesis. Leaf senescence further facilitated this remobilization by promoting the degradation of nitrogenous compounds and accelerating N export from leaves. Consequently, a lower SLN value indicated a diminished capacity for N accumulation per unit leaf area. Poorter et al. (2009) reported that the anatomical structure of leaves was substantially altered under water deficit conditions. In our study, the W0 treatment under rainout shelter conditions, characterized by prolonged water deficit, induced noticeable structural changes in leaf cells, including reduced cell size, thicker cell walls, and increased lignin content (Roig-Oliver et al., 2021; Zahedi et al., 2025). These structural modifications could limit the capacity for N uptake and storage within the leaf tissues. Supporting this, Islam et al. (2022) reported that nitrates were primarily stored in the vacuoles, suggesting that such structural changes under water deficit may directly affect N storage in leaves.

Overall, our findings aligned well with previous studies in several key aspects. Consistent with the observations of Poorter et al. (2010 and 2012), we found that SLA was more responsive to water deficit than LBF, confirming the greater plasticity of SLA under varying soil moisture conditions. The observed reduction in SLN during grain filling stage also corroborated the well-established N remobilization processes described by Lemaire et al. (2008). However, our study advanced previous research by quantitatively decomposing PNC and LNC into their morphological and physiological components (LBF, SLA, and SLN) under multi-year, controlled water and N coupling treatments. This approach provided a more detailed understanding of how these component traits interact to influence N dynamics in winter wheat, an aspect that has received limited attention in earlier studies.

### 4.3 Analysis of the combined effect of water and nitrogen on the relationships between plant nitrogen concentration and leaf nitrogen concentration, and between plant nitrogen accumulation and leaf area index

The relationship between PNC and LNC revealed that LNC decreased as PNC declined during the growth of winter wheat (**Figure 6A**). Changes in LNC were jointly regulated by both PNC and the ratio between SLN and LBF. Biologically, Equation 6 illustrated that LNC was influenced not only by PNC but also by the relative distribution of N and biomass within the plant. Specifically, LNC increased when the SLN-to-LBF ratio was high, indicating greater N accumulation per unit leaf area and a larger proportion of biomass allocated to leaves. Thus, LNC reflected both the plant’s N status and the efficiency of N partitioning and accumulation within the leaf tissues. Previous studies have shown that the extent of N dilution in leaves was lower than that at whole-plant level (Yao et al., 2014a, b; Sieling and Kage, 2021), which could be explained by the ratio between SLN and LBF. Due to the allometric relationship between SLN and LBF, the proportional decline in SLN was less pronounced than that of LBF during the growth of winter wheat (**Figure 6B**). SLN remained relatively stable before grain filling stage (**Figure 4**), whereas LBF showed a more rapid decline from stem elongation to anthesis (**Figure 2**). Consequently, the SLN-to-LBF ratio increased from stem elongation to anthesis. According to Equation 6, there was a trade-off between the increasing SLN to LBF ratio and the decreasing PNC as winter wheat developed, which ultimately determined the extent of leaf N dilution. This trade-off led to a more gradual decline in LNC compared to PNC.

The proportional linear relationship between NA_p_ and LAI was validated across different N and water coupling treatments in this study (**Figure 5A**). Water deficit did not affect the stability of this proportional linear relationship, which was consistent with the findings of Lemaire et al. (2008). Similarly, the relationship between NA_L_ and LAI also exhibited an approximately proportional linear pattern under different N and water coupling treatments. This could be attributed to the relatively stable SLN values observed from stem elongation to anthesis under the same treatment conditions. However, this result differed from that reported by Lemaire et al. (2007). In the present study, the value of parameter *a* (13.41) was significantly lower than the value (26.3) reported by Lemaire et al. (2007). This discrepancy might be due to the inclusion of data from all water and N coupling treatments (ranging from LNW0 to HNW3), where lower NA_L_ values under LN conditions reduced the overall estimate of parameter *a*. Conversely, the value of parameter *b* (1.1) in this study was significantly higher than the value (0.69) reported by Lemaire et al. (2007), potentially due to differences in cell volume between severe and non-severe water treatments. Since vacuolar size was closely associated with cell volume in plants, and vacuoles were key organelles involved in turgor-dependent cellular regulation (Dünser et al., 2022), cell enlargement primarily occurs through water uptake driven by the osmotic potential within cells. The accumulation of water in vacuoles increased their volume (Kruger and Schumacher, 2018). Under non-severe or normal water treatments, vacuole size was typically larger (Wu et al., 2024), allowing for greater N accumulation within the vacuole. Adequate water supply also increased LAI, and as shown in **Figure 5B**, this increase in LAI was associated with enhanced N accumulation in the leaf.

## 5 Conclusion

The reasons behind the decline in NNI were analyzed from the perspective of PNC, based on the NNI calculation equation. A significant decline in both PNC and LNC was observed at specific growth stages of winter wheat. The decline in PNC was attributed to reductions in LBF and SLA, whereas the decline in LNC was mainly due to changes in SLA and SLN. However, the water status under rainout shelter conditions was unlikely to replicate typical field conditions; therefore, this study suggested that PNC was not the primary cause of the NNI decline under field water deficit conditions. The change in LNC could be explained by the trade-off between PNC and the ratio of SLN to LBF. From a practical perspective, it is essential to consider the crop’s water status when calculating NNI to achieve a more accurate assessment of the crop’s N status. This approach will help in better N management, enhance nutrient use efficiency, and promote crop production.

## Data Availability

The raw data supporting the conclusions of this article will be made available by the authors, without undue reservation.

## Supplementary material

Supplementary Table 1 Detailed information of irrigation amount at different growth stages of winter wheat.
