# Supplementary material for: Exploring the effect of plant nitrogen concentration on the nitrogen nutrition index of winter wheat under controlled irrigation conditions
Source: Front Plant Sci. 2025 Jul 1;16:1609847. doi: 10.3389/fpls.2025.1609847 (PMC12260536; doi:10.3389/fpls.2025.1609847)
Supplement: Supplementary Table 1 — Detailed information of irrigation amount at different growth stages of winter wheat. [file Table1.pdf]

Supplementary Table S1 the detailed information of irrigation amount at different growth stages of winter wheat.

Experiments	Water levels	Irrigation amount (mm) at different growth stages				
		Sowing	Over-wintering	Stem elongation	Booting	Filling
Exp. 1	W0	60	60	0	0	0
	W1	60	60	30	30	30
	W2	60	60	60	60	60
	W3	60	60	90	90	90
Exp. 2	W0	60	180	0	0	0
	W1	60	180	30	30	30
	W2	60	180	60	60	60
	W3	60	180	90	90	90
Exp. 3	W0	60	60	0	0	0
	W1	60	60	30	30	30
	W2	60	60	60	60	60
	W3	60	60	90	90	90
